# Cancer stem cells in head and neck squamous cell carcinoma

**DOI:** 10.1186/1753-6561-7-S2-P14

**Published:** 2013-04-04

**Authors:** Christina Karamboulas, Keira Pereira, Elzbieta Hyatt, Laurie E Ailles

**Affiliations:** 1 Ontario Cancer Institute, University Health Network, Toronto, Canada; 2 Department of Medical Biophysics, University of Toronto, Toronto, Canada

## Background

Head and Neck Squamous Cell Carcinomas (HNSCCs) are tumors that display a high degree of heterogeneity. HNSCCs contain tumor-initiating cells (TICs) or cancer stem cells (CSCs) that are enriched within the CD44+ fraction and are capable of initiating and maintaining tumor growth. HNSCCs also contain a stromal component, consisting of cancer associated fibroblasts (CAFs), endothelial cells, and immune cells, which also play an important role in tumor progression. This heterogeneity can obscure results and thus obtaining pure populations of TICs, non-TICs and CAFs is essential for more stringent analysis.

## Materials and methods

HNSCC specimens were dissociated into single cell suspensions, stained for cell surface markers and examined by fluorescence-activated cell sorting. A high-throughput screen for 340 cell surface markers was used to identify candidate biomarkers that could further enrich for TICs that were then functionally tested by performing in vivo limiting dilution assays. Total RNA was extracted from isolated TIC, non-TIC and CAF populations and real time PCR was performed.

## Results

Three candidate biomarkers were identified and one marker, CD271, resides within the CD44+ fraction and further enriches for TIC activity. In well-differentiated tumors CD271 consistently localizes to the basal layer. Moreover, TIC, non-TIC and CAF populations contain distinct expression profiles.

## Conclusions

We have identified CD271 as a biomarker that enriches for TIC activity. The distinguishing expression profiles for TICs, non-TICs, and CAFs are being further analyzed to determine essential signaling cascades that exist among these populations that could potentially be targeted for HNSCC treatment.

